# Ischemia and Reperfusion Induce Differential Expression of Calpastatin and Its Homologue High Molecular Weight Calmodulin-Binding Protein in Murine Cardiomyocytes

**DOI:** 10.1371/journal.pone.0114653

**Published:** 2014-12-08

**Authors:** Sreejit Parameswaran, Rajendra K. Sharma

**Affiliations:** Department of Pathology and Laboratory Medicine, College of Medicine, University of Saskatchewan, Saskatoon, Saskatchewan, Canada; University Hospital Medical Centre, Germany

## Abstract

In the heart, calpastatin (Calp) and its homologue high molecular weight calmodulin-binding protein (HMWCaMBP) regulate calpains (Calpn) by inhibition. A rise in intracellular myocardial Ca^2+^ during cardiac ischemia activates Calpn thereby causing damage to myocardial proteins, which leads to myocyte death and consequently to loss of myocardial structure and function. The present study aims to elucidate expression of Calp and HMWCaMBP with respect to Calpn during induced ischemia and reperfusion in primary murine cardiomyocyte cultures. Ischemia and subsequently reperfusion was induced in ∼80% confluent cultures of neonatal murine cardiomyocytes (NMCC). Flow cytometric analysis (FACS) has been used for analyzing protein expression concurrently with viability. Confocal fluorescent microscopy was used to observe protein localization. We observed that ischemia induces increased expression of Calp, HMWCaMBP and Calpn. Calpn expressing NMCC on co-expressing Calp survived ischemic induction compared to NMCC co-expressing HMWCaMBP. Similarly, living cells expressed Calp in contrast to dead cells which expressed HMWCaMBP following reperfusion. A significant difference in the expression of Calp and its homologue HMWCaMBP was observed in localization studies during ischemia. The current study adds to the existing knowledge that HMWCaMBP could be a putative isoform of Calp. NMCC on co-expressing Calp and Calpn-1 survived ischemic and reperfusion inductions compared to NMCC co-expressing HMWCaMBP and Calpn-1. A significant difference in expression of Calp and HMWCaMBP was observed in localization studies during ischemia.

## Introduction

Cardiovascular disease is the leading cause of morbidity and mortality in the world despite the improvements in prevention, detection and treatment [1]. In general, artery blockage results in cardiac ischemia due to reduction of the blood supply to cardiac muscle. This event causes oxygen and nutrient deprivation and the buildup of toxic products [2]. Prompt reperfusion (restoration of blood flow) limits the damage and reduces mortality [3]. Ironically however, additional cardiac damage and complications are often the consequences with the return of blood flow, a clinical condition termed reperfusion injury [4]. During cardiovascular disorders, increase in Ca^2+^ activates signaling cascades leading to hypertrophy and cell death especially through the activation of various kinases and phosphatases [5], [6]. In the heart, the key proteins such as calmodulin (CaM), calpains (Calpn), calcineurin (CaN), calpastatin (Calp), and phosphodiesterase-1 are regulated by Ca^2+^ [7]. These proteins act in a regulated and concerted manner for the proper functioning of heart muscle. Not much is known about the regulation and interaction among these proteins and associated molecules during cardiac injury caused by ischemia and reperfusion (I/R) [8]–[11].

Calpains are Ca^2+^-activated cysteine proteases present in the cytosol as inactive proenzymes [10]. Calp is the most efficient and specific calpain inhibitor present *in vivo* [9]. Earlier, we reported the high expression of high molecular weight calmodulin-binding protein (HMWCaMBP) in human and animal cardiac tissues [9], [12]. HMWCaMBP showed calpastatin activity and was also found to be highly homologous to calpastatin I and calpastatin II [13], [14]. A decreased expression of HMWCaMBP was observed during ischemia due to its susceptibility to proteolysis by calpains during I/R [15]. In normal myocardium, HMWCaMBP may protect its substrate from calpains. However during I/R, due to increased Ca^2+^ influx, calpain activity often exceeds HMWCaMBP activity [8], [16]. This leads to proteolysis of HMWCaMBP and other protein substrates, resulting in cellular damage. The role of Calp and its homologue HMWCaMBP in I/R and their interactions are not completely elucidated [9]. In our previous report, this assay helped in determining cells which can survive I/R injury and most importantly the proteins responsible for the same [8]. Previous studies showed that HMWCaMBP and Calp interact with Calpn and regulates degradation of cellular proteins which results in the death of cardiac cells following I/R [8]–[10], [12]–[16].

In the present study HMWCaMBP, a homologue of Calp with calmodulin (CaM)-binding property and the ability to inhibit Calpn, was prioritized and expression levels were compared to Calp [8], [9], [13]–[16]. Furthermore, the current study aims to elucidate the differential expression of Calp and HMWCaMBP in cardiomyocytes following I/R using flow cytometric analysis (FACS). The altered expression levels of Calp and its homologue HMWCaMBP in relation to live-dead analysis can help us to predict which cells will be able to survive the I/R insult. By using co-localization studies, the current study aims to identify whether HMWCaMBP is an isoform of Calp and could be designated as Calp-4.

## Methods

### Isolation and culture of neonatal murine cardiomyocytes

Neonatal murine cardiomyocyte culture (NMCC - primary culture derived from murine heart) was used for studying induced I/R injury. 2-8 day old CD-1 Swiss albino mice pups were sacrificed in accordance to the protocol (Animal Use Protocol # 20120011) approved by the University of Saskatchewan Animal Research Ethics Board. The pups were guillotined and the beating hearts were immediately removed. Cardiomyocytes were isolated and cultured on 0.02% gelatin-precoated cell culture flasks as per the protocol described previously [17], [18]. Compared to our previous study [8], an additional differential plating step was performed to increase the number of isolated cardiomyocytes. The primary cultures were maintained till the cultures attained ∼80% and then the cells were induced with I/R injury.

### Induction of ischemia and reperfusion

The media in NMCC cultures (∼80% confluent) was replenished 24 hrs prior to induction. Ischemia was induced by replacing the media with a nutrient deficient buffer (NDB) and subsequently, reperfusion was carried out by replacing NDB with standard growth media [19]–[21]. The methodology as described in S1 Methods was performed as per a previously published protocol [8].

### Assessment of protein expression and viability

Simultaneous assessment of protein expression in control, ischemic and reperfused cardiomyocytes along with viability was performed by FACS. The methodology was performed as per a previously published protocol [8]. The antibodies used have been tabulated along with the dilutions used in S1 Table.

### Microscopy

Fluorescent and subsequently confocal microscopy was performed to demonstrate the protein expression in control, ischemic and reperfused cardiomyocytes. The methodology as described in the S1 Methods was optimized by staining cells with primary antibodies and subsequently with appropriate fluorophore conjugated secondary antibodies and observed under a fluorescent microscope. The antibodies used for the study have been tabulated along with the dilutions used in S1 Table.

### Statistical analysis

Statistical analysis was performed using the Student t-test from the data obtained from the various assays with the Sigma Plot version 10 software package. Statistical significance of co-localization studies was calculated using ANOVA and compared with the Holm-Sidak method (significance level = 0.05). p-values were calculated and represented as * - <0.05; **-<0.001; *** - <0.0001, to indicate significant differences.

## Results and Discussion

### Comparative expression of Calp, HMWCaMBP and α-sarcomeric actin with Calpn-1 by triple staining

**Figure 1:**
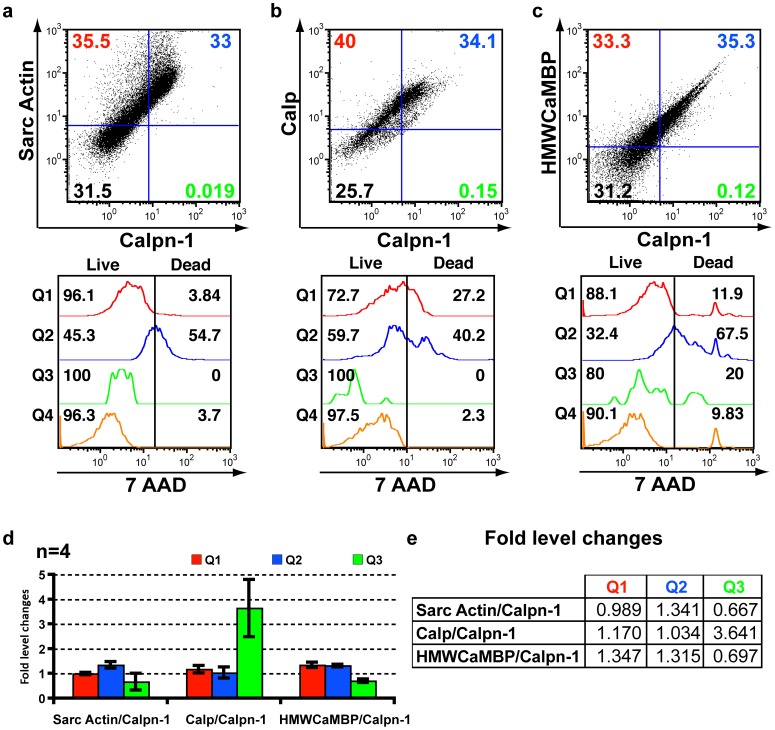
FACS data of NMCC following ischemia. (a-c) Representative FACS data of NMCC following ischemia induction along with a live-dead assay. In the vertical axis, PE labeled antibodies against α-sarcomeric actin (Sarc Actin) (Fig. 1a), calpastatin (Calp) (Fig. 1b) and high molecular weight calmodulin-binding protein (HMWCaMBP) (Fig. 1c) and for the horizontal axis FITC labeled anti-Calpain-1 (Calpn-1) antibodies were detected. The remaining figures in the panel (Q1–Q4) are derived from the quadrants of the Fig. 1 (a-c) and demonstrate live-dead assay using 7-AAD. NMCC treated with nutrient deficient buffer (ischemia induction –S1 Methods) for 2 h. (d) Comparison of ischemia induced average protein expression in NMCC with those of control NMCC (normal untreated –S2 Figure) (quadrants with staining-Q1–Q3 only) represented as fold level change in a histogram (n = 4). The fold level increase or decrease of protein expressing NMCC in each quadrant has been represented. (e) Tabulated representation of fold level changes in ischemia induced protein expression in NMCC within stained quadrants (Q1–Q3) in comparison with control cells (n = 4).

**Figure 2:**
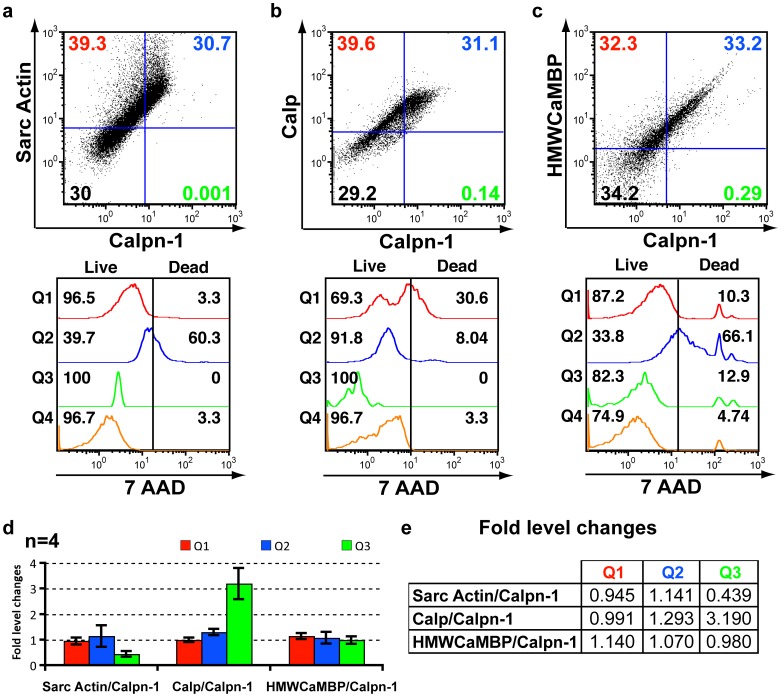
FACS data of NMCC following reperfusion. (a-c) Representative FACS data of NMCC following reperfusion induction along with a live-dead assay. In the vertical axis, PE labeled antibodies against α-sarcomeric actin (Sarc Actin) (Fig. 2a), calpastatin (Calp) (Fig. 2b) and high molecular weight calmodulin-binding protein (HMWCaMBP) (Fig. 2c) and for the horizontal axis FITC labeled anti-Calpain-1 (Calpn-1) antibodies were detected. The remaining figures in the panel (Q1–Q4) are derived from the quadrants of the Fig. 2 (a-c) and demonstrate live-dead assay using 7-AAD. NMCC grown for 2 h in normal media containing 1 mM H_2_O_2_ following 2 h of ischemia induction (reperfusion induction – S1 Methods). (d) Comparison of ischemia induced average protein expression in NMCC with those of control NMCC (normal untreated – S2 Figure) (quadrants with staining-Q1–Q3 only) represented as fold level change in a histogram (n = 4). The fold level increase or decrease of protein expressing NMCC in each quadrant has been represented. (e) Tabulated representation of fold level reperfusion induced protein expression in NMCC within stained quadrants (Q1–Q3) in comparison with control cells (n = 4).

The standardization of I/R treatments along with the expression of various cardiac proteins in living and dead cells were analyzed in control and induced NMCC as previously described [8]. Triple staining was performed simultaneously for two proteins of interest with specific antibodies tagged with the fluorophore (FITC and PE, respectively) along with a live-dead assay of stained cells with 7AAD. The assay specifically meets the aim of elucidating the cardiac protein expression in cells following ischemia and subsequent reperfusion. On comparing the expression of α-sarcomeric actin (Sarc Actin), Calp and HMWCaMBP with Calpn-1 in normal, ischemia induced and reperfusion induced cells, we observed an increased expression of both Calp and its homologue HMWCaMBP following ischemia (Fig. 1), which partially reverted back to normal levels following reperfusion (S1 Figure). The data are consistent with previous reports on the expression of both proteins [8], [15], [16]. A live-dead assay using 7-AAD demonstrated that the expression of Calp and HMWCaMBP in living cells decreased following ischemia and increased during subsequent reperfusion [8]. The percentage of Calpn-1 expressing living cardiomyocytes decreased following ischemia; however subsequent to reperfusion, the percentages returned back to normal levels (pre-ischemia induction controls) when compared with Calp (S2 Figure). A significant decrease in living cells expressing HMWCaMBP was observed which decreased following ischemia, and subsequently reperfusion barely increased the number of living cells expressing HMWCaMBP (Fig. 1c). The expression of Sarc Actin, constitutively expressed actin in cardiomyocytes, remained consistent in normal (S2 Figure) and I/R induced cardiomyocytes (Figs. 1 and 2). The number of living cardiomyocytes expressing Sarc Actin decreased following ischemia and subsequently reperfusion (Figs. 1a and 2a). Similarly, the number of living Calpn-1 expressing cells decreased following I/R induction (Figs. 1 and 2).

### Expression studies using confocal fluorescence microscopy

**Figure 3:**
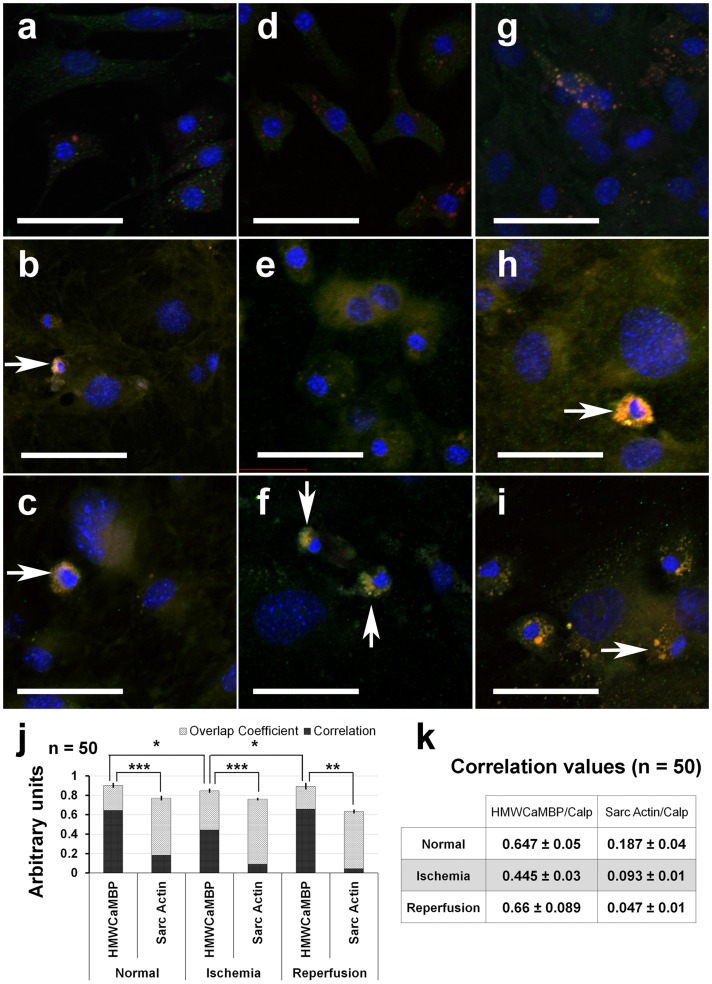
Confocal microscopy of NMCC. Confocal microscopy images of NMCC – (a,d,g) control; (b,e,h) ischemia induced; (c,f,i) reperfusion induced (Scale Bar – 50 µm). NMCC were stained with PE labeled antibodies against calpastatin (a-c), high molecular weight calmodulin-binding protein (HMWCaMBP) (d-f) and Alexa Flour 488 labeled anti-Calpain-1 antibodies (a-e). PE labeled antibodies against HMWCaMBP and Alexa Flour 488 labeled anti-Calp were also used to stain NMCC (g-i). DAPI in SlowFade Gold antifade reagent stains nucleus in cells (a-i). White arrowhead in the images indicate dead cells (nuclear condensation), where enhanced calpastatin (b,c,h,i) and HMWCaMBP (f,h,i) expression was observed along with calpain-1 (b,c,f). (j) Histographical representation of the overlap coefficient and correlation values of HMWCaMBP and α-sarcomeric actin (Sarc Actin) in comparison with Calp. p-values were calculated from the correlation values in cells (n = 50) and represented in the figure as * - <0.05; **-<0.001; *** - <0.0001. (k) Tabulated values of correlation between HMWCaMBP and Calp in comparison to α-sarcomeric actin (Sarc Actin) and Calp during ischemia and reperfusion.

The expression of Calp and its homologue HMWCaMBP along with Calpn-1 increased during ischemia (Fig. 3b,e,h). Calp expression remained almost consistent following reperfusion (Fig. 3c and S3f Figure). However, reperfusion resulted in decreased HMWCaMBP expression (Fig. 3f). It was also observed that the expression of both Calp and HMWCaMBP remained increased in dead cells (as denoted by arrows in Fig. 3c,f,i). Expression of Calpn-1 decreased slightly during reperfusion; however the expression remained consistent in dead cells (Fig. 3c,f,i and S4d-f Figure). Sarc Actin expression which by nature is constitutive and is not related to Calpn activation during I/R, has no effect on the co-expressing Calp (as observed in S1 and S5 Figures). Co-localization analysis demonstrated a significant difference in localization of HMWCaMBP and Calp in NMCC during ischemia in comparison to normal and reperfused NMCC (Fig. 3j). Although similar overlap coefficient values were observed on comparing Sarc Actin and Calp, the correlation values were significantly lower than HMWCaMBP and Calp in NMCC (Fig. 3k).

### HMWCaMBP: an isoform of Calp

On comparing HMWCaMBP with Calp, it was observed that ischemia increases the expression of HMWCaMBP in general with a simultaneous increase in Calp expression (Fig. 3g-i and S5 Figure). This was followed by a significant decrease in HMWCaMBP expression compared to a minimal decrease in Calp expression during reperfusion. Calp on the other hand, responds to an increase in Calpn [22] and is not affected by the changes in [Ca^2+^]i. An interesting FACS analysis observation was that of the increase in Calp expression in general due to I/R; however the number of Sarc Actin^+^ cardiomyocytes expressing Calp decreases slightly during ischemia and subsequent reperfusion (S4 Figure). Activation of Calpn is often followed by the increase in Calp expression (Fig. 1b) and the expressed Calp inhibits the majority of activated Calpn [8], [16]. The slight delay in increase of Calp compared to HMWCaMBP during ischemia could have increased the likelihood of cells expressing Calp compared to HMWCaMBP. A similar pattern is observed even during reperfusion where the chances of cells expressing Calp surviving the stress due to reperfusion injury are increased in comparison to cells expressing HMWCaMBP (Fig. 2).

The contrast in expression of Calpn-1 in cardiomyocytes in comparison to Sarc Actin, Calp and HMWCaMBP as observed by FACS analysis is directly related to the protein being studied. Calpn-1 activation requires micromolar amounts of intercellular calcium ([Ca^2+^]i) [23]. α-sarcomeric actin does not regulate [Ca^2+^]i and therefore, does not influence the expression of Calpn-1. This is observed as an increase in the number of dead cardiomyocytes expressing Calpn-1 during reperfusion since Calpn-1 activity has been implicated in I/R injury [24]. Calp and HMWCaMBP sequester Calpn from its substrates in the normal myocardium, but may be proteolyzed during the early phase of Calpn activation during I/R. Calpn activation results in the proteolysis of Calp and HMWCaMBP followed by other calpain substrates [9], [13], [22], [25]. Besides, HMWCaMBP can regulate Calpn activity through Ca^2+^ [9]. The binding of Calp and HMWCaMBP to Calpn results in decreased activity in a shorter time scale [22]. The longer I/R induction used in this study results in expression of Calp and HMWCaMBP to compensate for the activity loss and minimize I/R induced stress and injury. An increase in Calp and HMWCaMBP effectively inhibits Calpn-1, thus reducing proteolysis and leading to cell survival. These interactions could be responsible for the increase in number of living cells expressing Calpn-1 during the process of reperfusion.

The difference in expression of Calp and HMWCaMBP by living cardiomyocytes during reperfusion could be due to the loss of Ca^2+^ homeostasis following ischemia. Ischemia causes a rise in [Ca^2+^]i which leads to the activation of various receptors, signaling molecules and proteins including CaM and Calpn [26], [27]. Since, HMWCaMBP has a CaM-binding site and becomes phosphorylated in the presence of Ca^2+^ and CaM [9], the rise in [Ca^2+^]i could involuntarily induce HMWCaMBP expression. Simultaneous Calpn activation could also result in the inhibition by HMWCaMBP during the initial stages of ischemia. The initial increase in expression of HMWCaMBP usually does not stem the simultaneous Calpn activation during ischemia often resulting in cell death [15], [16]. The above results are in agreement to our previous studies suggesting that HMWCaMBP besides being a homologue of Calp, could also be a putative isoform of Calp [13]–[16]. Confocal studies in NMCC with HMWCaMBP and Calp show a similar expression (Fig. 3g-i). However, the altered co-localization of HMWCaMBP and Calp during ischemia suggests that HMWCaMBP may be a isoform of Calp. Furthermore, HMWCaMBP can also be potentially used as a marker to determine cells which have a lesser chance of survival following ischemia and subsequent reperfusion [8]. However to prove whether HMWCaMBP is an isoform of Calp, further studies need to be carried out by the use of knockout models.

It should be noted that the present study is similar and replicative to our previous report [8] related to the expression of HMWCaMBP with Calpn-1 and Calp using FACS. However, an entirely new set of FACS data was used in this study to demonstrate the observations (Figs. 1 and 2 and S2 and S3 Figures). This is because of the improvement in the isolation protocol for cardiomyocytes from mouse hearts which resulted in a drastic increase in number of isolated cardiomyocytes (S6 Figure). This resulted in a more precise observation in obtaining the expression of HMWCaMBP, Calpn-1 and Calp. Furthermore, the replicated FACS data are required to compare and validate the expression of HMWCaMBP and Calp with the constitutively expressed Sarc Actin.

## Conclusions

The present study describes the role of Calp and its homologue HMWCaMBP in I/R and their interactions with Calpn-1 in normal, ischemia-induced and reperfused cardiomyocytes. The overall percentage of Calpn-1 expressing cells increased with ischemic induction and reduced following reperfusion; however the number of living cells expressing Calpn-1 decreased during ischemia and reverted to normalcy after reperfusion. The expression of Calp and its homologue HMWCaMBP increased following ischemia and then decreased following reperfusion. However, the Calpn-1 expressing cells on co-expressing Calp were predominantly alive in comparison to the dead cells co-expressing HMWCaMBP. The overall expression of both Calp and HMWCaMBP slightly increased during ischemia and partially reverted back to pre-ischemic levels following reperfusion. Calp expressing living cells were much higher than in those expressing HMWCaMBP during reperfusion due to the expression of Calpn during I/R. The reduction of HMWCaMBP expression in living cells following ischemia and reperfusion suggests poor survival outcome of cells expressing HMWCaMBP. Hence, HMWCaMBP can be potentially used as a marker to detect cells predestined towards cell death. The above study adds to the current knowledge which suggests that HMWCaMBP may be a putative isoform of Calp.

## Supporting Information

S1 Figure **Comparative expression of Sarc Actin, Calp and HMWCaMBP with Calpn-1.** (a) Comparison of reperfusion induced average protein expression in NMCC (Fig. 2) with those of ischemia induced NMCC (Fig. 1) (quadrants with staining - Q1–Q3 only) represented as a histogram (n = 4). The percentage increase or decrease of protein expressing NMCC in each quadrant has been represented. (b) Tabulated representation of fold level changes in reperfusion induced protein expression in NMCC within stained quadrants (Q1–Q3) in comparison with ischemia induced protein expression in NMCC.
(TIF)

S2 Figure **Representative FACS data of NMCC in absence of I/R induction along with live-dead assay.** (a-c) In the vertical axis, PE labeled antibodies against α-sarcomeric actin (Sarc Actin) (a), calpastatin (Calp) (b) and high molecular weight calmodulin-binding protein (HMWCaMBP) (c) and for the horizontal axis FITC labeled anti-Calpain-1 (Calpn-1) antibodies were detected. The remaining figures in the panel (Q1–Q4) are derived from the quadrants of the S2 Figure (a-c) and demonstrate live-dead assay using 7-AAD. NMCC were grown and maintained in standard cardiomyocyte maintenance media without any treatment for obtaining control cells.
(TIF)

S3 Figure **Confocal microscopy images of NMCC.** (a,d) control; (b,e) ischemia induced; (c,f) reperfusion induced (Scale Bar – 50 µm). NMCC were stained with PE labeled antibodies against calpain-1 (a-c), calpastatin (d-f) and Alexa Flour 488 labeled anti-α-sarcomeric actin antibodies (a-e). DAPI in SlowFade Gold antifade reagent which stains nuclei in cells (a-i). White arrowhead in the images indicate dead cells (nuclear condensation), where enhanced calpain-1 (b,c) and calpastatin (f) expression were observed.
(TIF)

S4 Figure **FACS of NMCC following I/R induction for α-sarcomeric actin and calpastatin.** (a-c) Representative FACS data of NMCC following I/R induction along with a live-dead assay. In the vertical axis PE labeled antibodies against α-sarcomeric actin (Sarc Actin) and for the horizontal axis FITC labeled anti- calpastatin (Calp) antibodies were detected. The remaining figures in the panel (Q1–Q4) are derived from the quadrants of the S4 Figure (a-c) and demonstrate live-dead assay using 7-AAD. The conditions used were; normal untreated NMCC – Ctrl (a); NMCC treated with nutrient deficient buffer (ischemia induction) for 2 h – I + O (b); NMCC grown for 2 h in normal media containing 1 mM H_2_O_2_ following 2 h of ischemia induction (reperfusion induction) – I + O + R (c). (d) Combined data of performed experiments (n = 4) (quadrants with staining - Q1–Q3 only) represented as percentage of cells in a histogram. The percentage of living and dead cells has been combined for each quadrant. (e) Tabulated representation of fold level changes of living cells in quadrants with staining (Q1–Q3) in comparison with normal control cells.
(TIF)

S5 Figure **FACS of NMCC following I/R induction for HMWCaMBP and calpastatin.** (a-c) Representative FACS data of NMCC following I/R induction along with a live-dead assay. In the vertical axis PE labeled antibodies against high molecular weight calmodulin-binding protein (HMWCaMBP) and for the horizontal axis FITC labeled anti- calpastatin (Calp) antibodies were detected. The remaining figures in the panel (Q1–Q4) are derived from the quadrants of the S5 Figure (a-c) and demonstrate live-dead assay using 7-AAD staining. The conditions used were; normal untreated NMCC – Ctrl (a); NMCC treated with nutrient deficient buffer (ischemia induction) for 2 h – I + O (b); NMCC grown for 2 h in normal media containing 1 mM H_2_O_2_ following 2 h of ischemia induction (reperfusion induction) – I + O + R (c). (d) Combined data of performed experiments (n = 4) (quadrants with staining - Q1–Q3 only) represented as percentage of cells in a histogram. The percentage of living and dead cells has been combined for each quadrant. (e) Tabulated representation of fold level changes of living cells in quadrants with staining (Q1–Q3) in comparison with normal control cells.
(TIF)

S6 Figure **Comparative live-dead assay of different cardiomyocyte isolation protocols.** The original protocol as described in previous study [1] was modified by including an additional differential plating step prior to final isolation of cardiomyocytes from heart tissue. The difference in the number of living and dead cells following isolation was assessed by 7AAD and the percentage of cells is represented as a histogram. p-values were calculated from the cell percentage values (n = 6) and represented in the figure as *** - <0.0001.
(TIF)

S1 Table **Antibodies and their dilutions used for FACS and Confocal Fluorescent Microscopy (CFM).**
(DOCX)

S1 Methods **Document describing methodology not provided in the manuscript.**
(DOC)
