# Supplementary figures and images for: Ischemia and Reperfusion Induce Differential Expression of Calpastatin and Its Homologue High Molecular Weight Calmodulin-Binding Protein in Murine Cardiomyocytes
Source: PLoS One. 2014 Dec 8;9(12):e114653. doi: 10.1371/journal.pone.0114653 (PMC4259361; doi:10.1371/journal.pone.0114653)

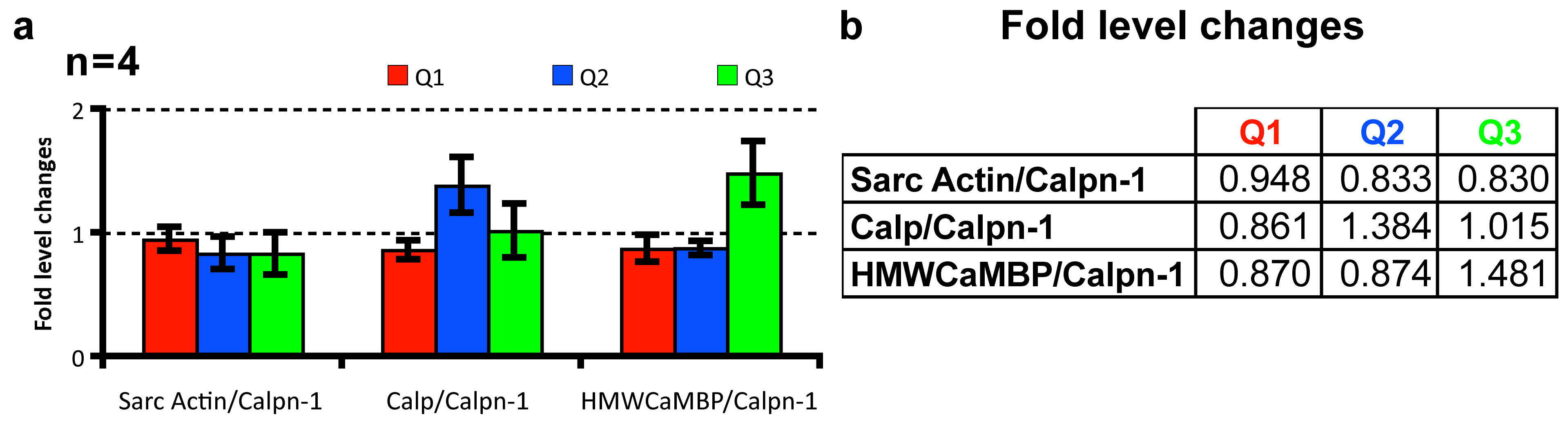

Supplement: S1 Figure — Comparative expression of Sarc Actin, Calp and HMWCaMBP with Calpn-1. (a) Comparison of reperfusion induced average protein expression in NMCC (Fig. 2) with those of ischemia induced NMCC (Fig. 1) (quadrants with staining - Q1–Q3 only) represented as a histogram (n = 4). The percentage increase or decrease of protein expressing NMCC in each quadrant has been represented. (b) Tabulated representation of fold level changes in reperfusion induced protein expression in NMCC within stained quadrants (Q1–Q3) in comparison with ischemia induced protein expression in NMCC. (TIF) [file pone.0114653.s001.tif]

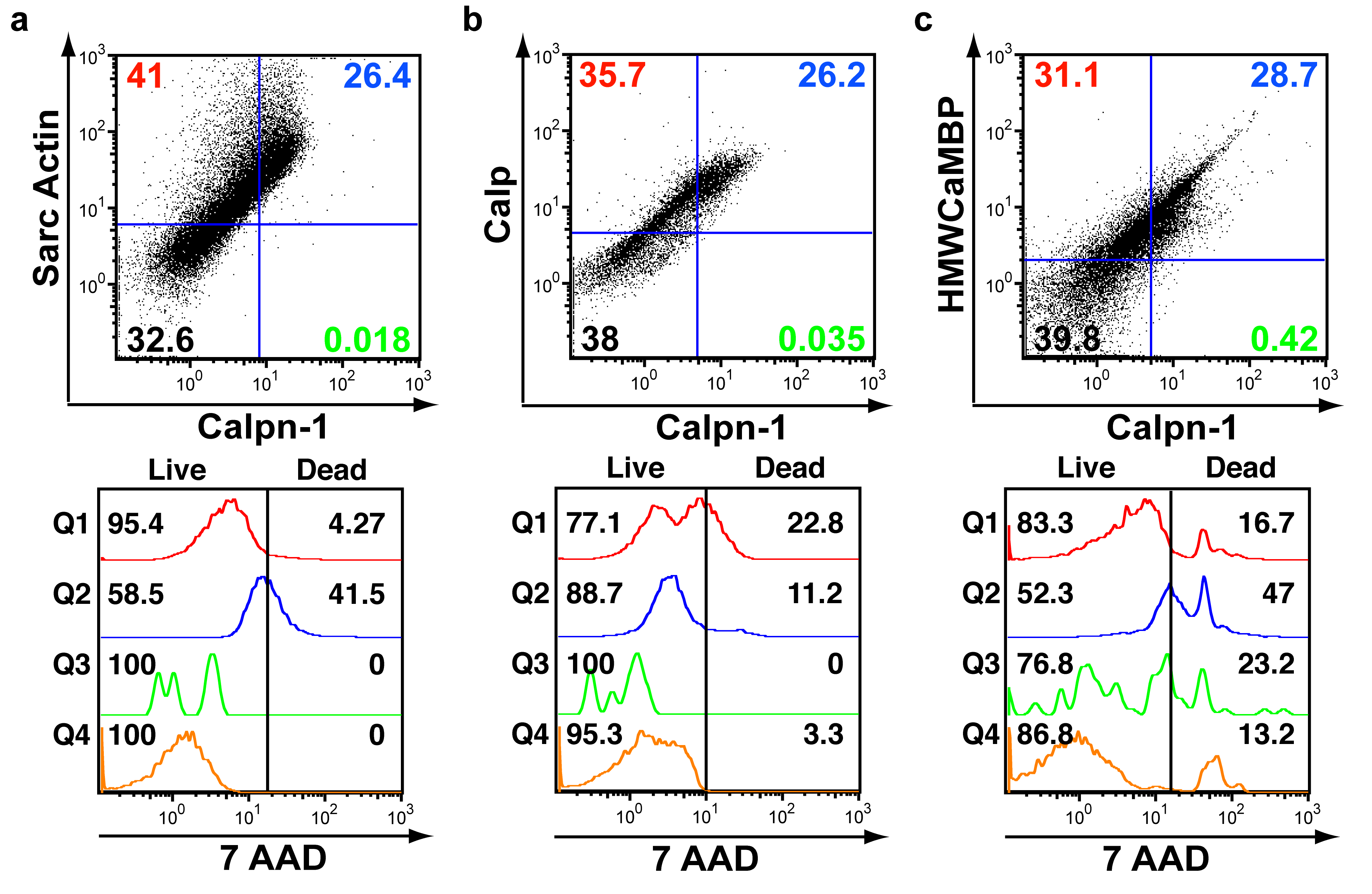

Supplement: S2 Figure — Representative FACS data of NMCC in absence of I/R induction along with live-dead assay. (a-c) In the vertical axis, PE labeled antibodies against α-sarcomeric actin (Sarc Actin) (a), calpastatin (Calp) (b) and high molecular weight calmodulin-binding protein (HMWCaMBP) (c) and for the horizontal axis FITC labeled anti-Calpain-1 (Calpn-1) antibodies were detected. The remaining figures in the panel (Q1–Q4) are derived from the quadrants of the S2 Figure (a-c) and demonstrate live-dead assay using 7-AAD. NMCC were grown and maintained in standard cardiomyocyte maintenance media without any treatment for obtaining control cells. (TIF) [file pone.0114653.s002.tif]

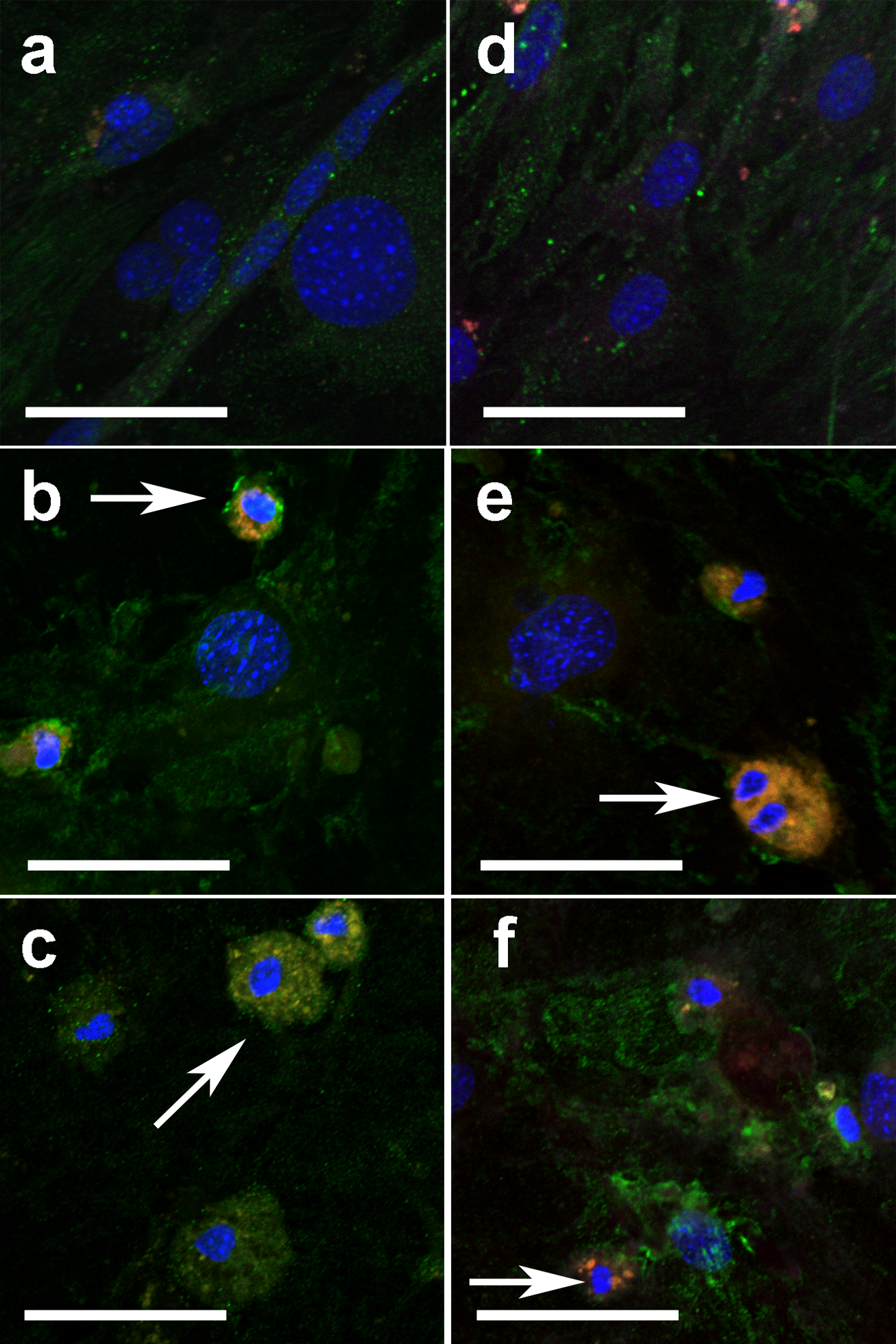

Supplement: S3 Figure — Confocal microscopy images of NMCC. (a,d) control; (b,e) ischemia induced; (c,f) reperfusion induced (Scale Bar – 50 µm). NMCC were stained with PE labeled antibodies against calpain-1 (a-c), calpastatin (d-f) and Alexa Flour 488 labeled anti-α-sarcomeric actin antibodies (a-e). DAPI in SlowFade Gold antifade reagent which stains nuclei in cells (a-i). White arrowhead in the images indicate dead cells (nuclear condensation), where enhanced calpain-1 (b,c) and calpastatin (f) expression were observed. (TIF) [file pone.0114653.s003.tif]

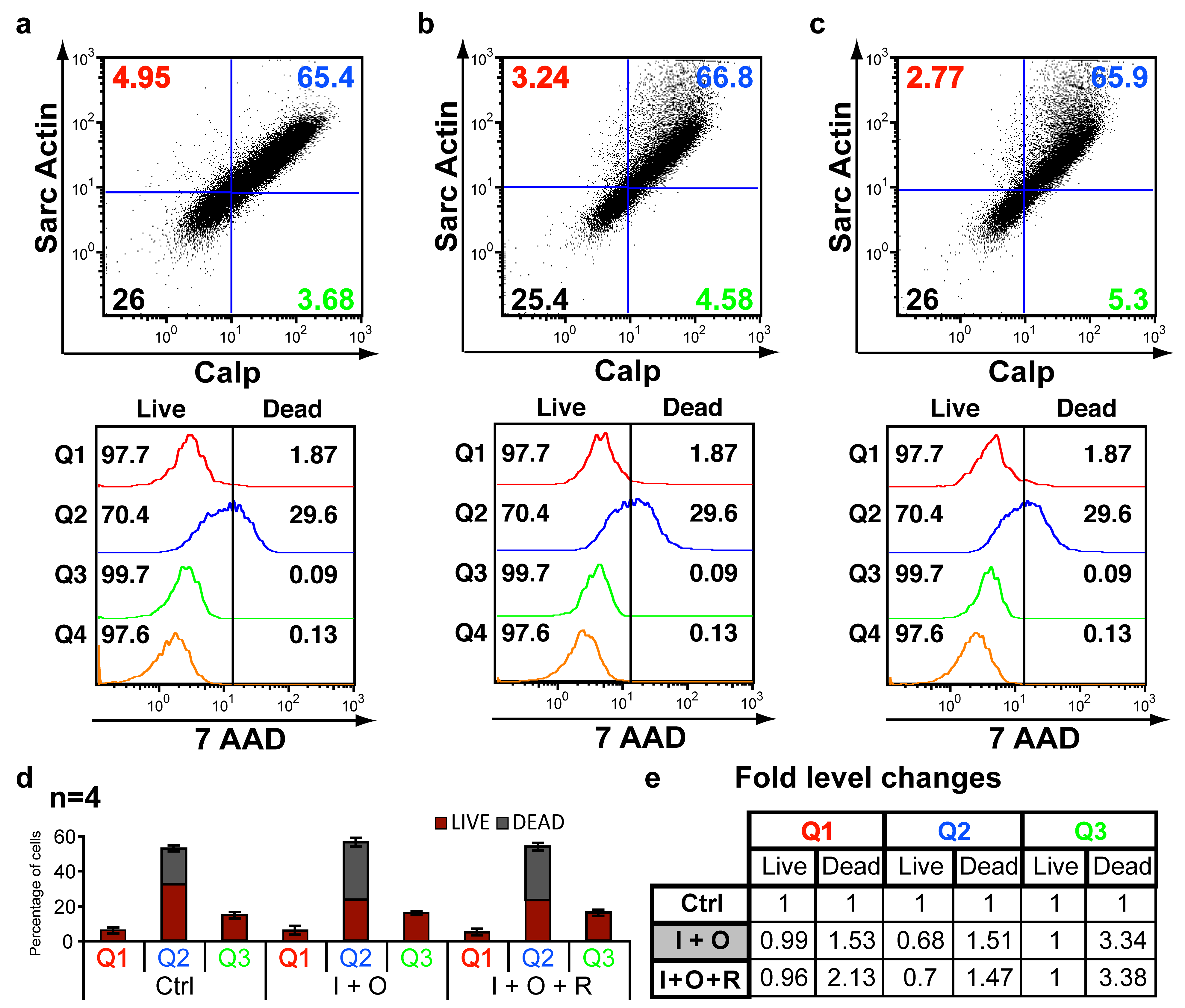

Supplement: S4 Figure — FACS of NMCC following I/R induction for α-sarcomeric actin and calpastatin. (a-c) Representative FACS data of NMCC following I/R induction along with a live-dead assay. In the vertical axis PE labeled antibodies against α-sarcomeric actin (Sarc Actin) and for the horizontal axis FITC labeled anti- calpastatin (Calp) antibodies were detected. The remaining figures in the panel (Q1–Q4) are derived from the quadrants of the S4 Figure (a-c) and demonstrate live-dead assay using 7-AAD. The conditions used were; normal untreated NMCC – Ctrl (a); NMCC treated with nutrient deficient buffer (ischemia induction) for 2 h – I + O (b); NMCC grown for 2 h in normal media containing 1 mM H2O2 following 2 h of ischemia induction (reperfusion induction) – I + O + R (c). (d) Combined data of performed experiments (n = 4) (quadrants with staining - Q1–Q3 only) represented as percentage of cells in a histogram. The percentage of living and dead cells has been combined for each quadrant. (e) Tabulated representation of fold level changes of living cells in quadrants with staining (Q1–Q3) in comparison with normal control cells. (TIF) [file pone.0114653.s004.tif]

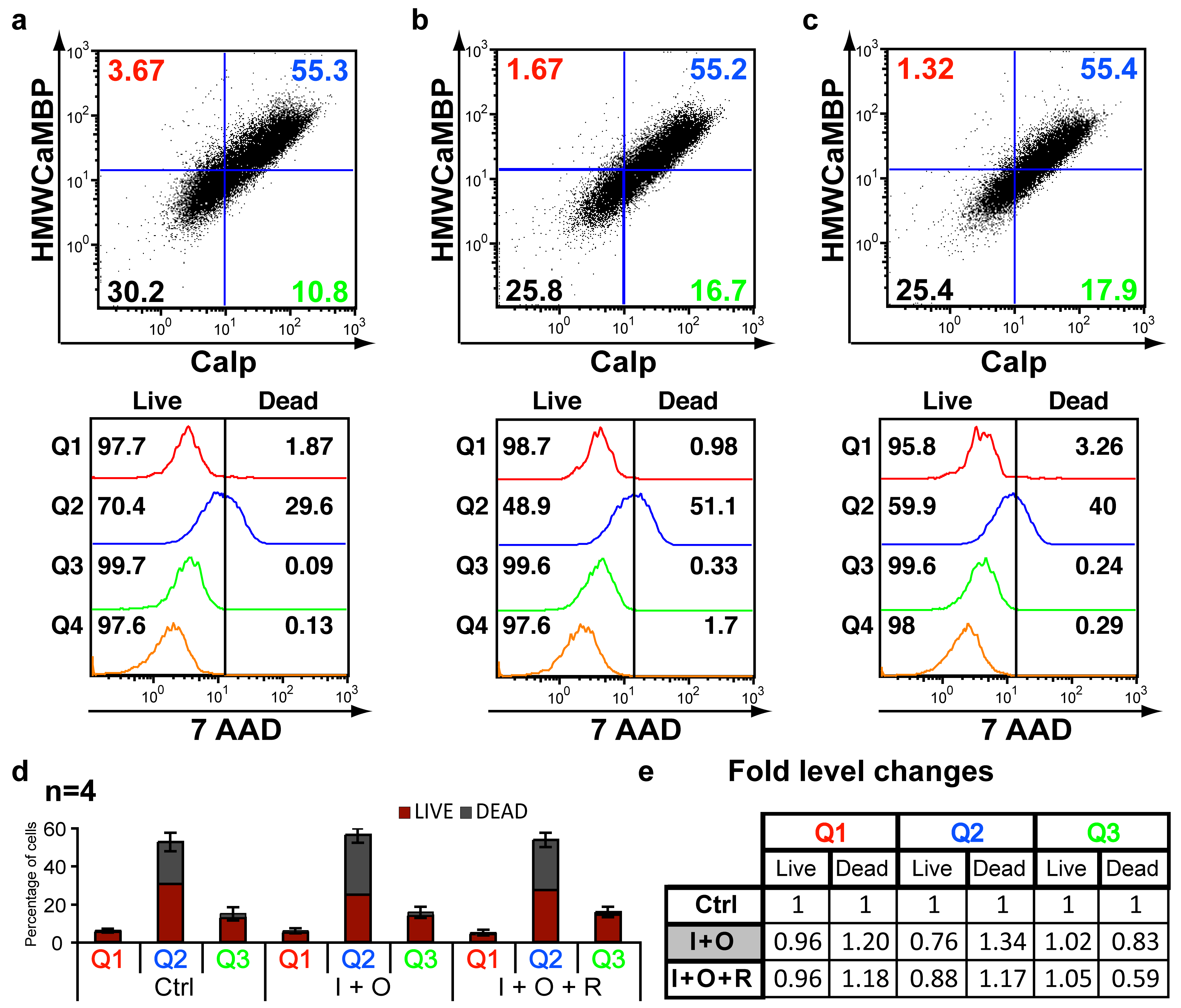

Supplement: S5 Figure — FACS of NMCC following I/R induction for HMWCaMBP and calpastatin. (a-c) Representative FACS data of NMCC following I/R induction along with a live-dead assay. In the vertical axis PE labeled antibodies against high molecular weight calmodulin-binding protein (HMWCaMBP) and for the horizontal axis FITC labeled anti- calpastatin (Calp) antibodies were detected. The remaining figures in the panel (Q1–Q4) are derived from the quadrants of the S5 Figure (a-c) and demonstrate live-dead assay using 7-AAD staining. The conditions used were; normal untreated NMCC – Ctrl (a); NMCC treated with nutrient deficient buffer (ischemia induction) for 2 h – I + O (b); NMCC grown for 2 h in normal media containing 1 mM H2O2 following 2 h of ischemia induction (reperfusion induction) – I + O + R (c). (d) Combined data of performed experiments (n = 4) (quadrants with staining - Q1–Q3 only) represented as percentage of cells in a histogram. The percentage of living and dead cells has been combined for each quadrant. (e) Tabulated representation of fold level changes of living cells in quadrants with staining (Q1–Q3) in comparison with normal control cells. (TIF) [file pone.0114653.s005.tif]

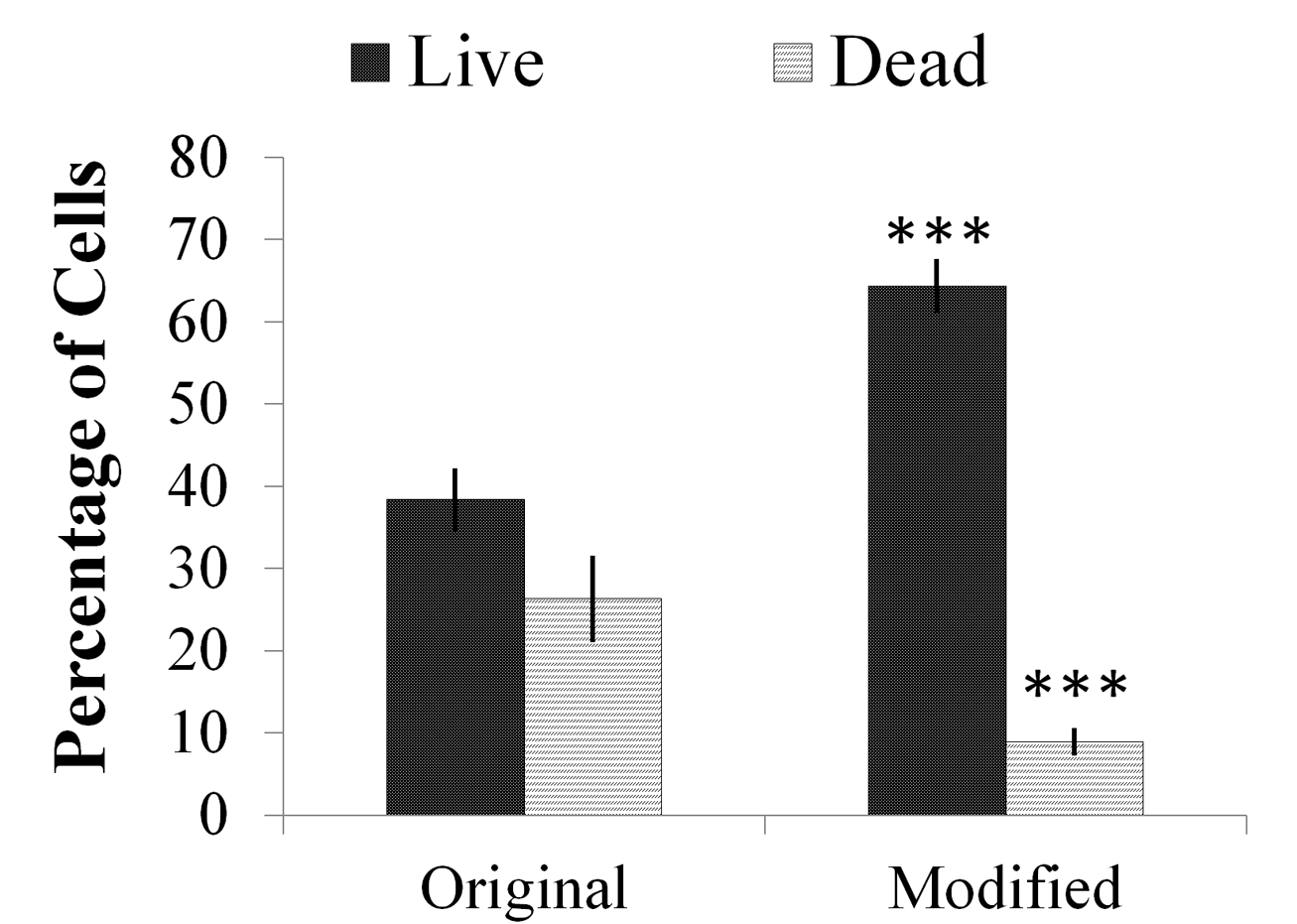

Supplement: S6 Figure — Comparative live-dead assay of different cardiomyocyte isolation protocols. The original protocol as described in previous study [1] was modified by including an additional differential plating step prior to final isolation of cardiomyocytes from heart tissue. The difference in the number of living and dead cells following isolation was assessed by 7AAD and the percentage of cells is represented as a histogram. p-values were calculated from the cell percentage values (n = 6) and represented in the figure as *** - <0.0001. (TIF) [file pone.0114653.s006.tif]
