# Supplementary material for: Ischemia and Reperfusion Induce Differential Expression of Calpastatin and Its Homologue High Molecular Weight Calmodulin-Binding Protein in Murine Cardiomyocytes
Source: PLoS One. 2014 Dec 8;9(12):e114653. doi: 10.1371/journal.pone.0114653 (PMC4259361; doi:10.1371/journal.pone.0114653)
Supplement: S1 Table — Antibodies and their dilutions used for FACS and Confocal Fluorescent Microscopy (CFM). (DOCX) [file pone.0114653.s007.docx]

**Supplementary Table S1** : Antibodies and their dilutions used for FACS and Confocal Fluorescent Microscopy (CFM)

| No. | **Antibody** | **Dilution used** | |
| --- | --- | --- | --- |
| **Primary antibodies** | | **FACS** | **CFM** |
| **1.** | Mouse anti mouse calpain-1 monoclonal antibodies (Thermo Sci.) | 1:500 | 1:200 |
| **2.** | Rabbit anti mouse α-sarcomeric actin polyclonal antibodies (Thermo Sci.) | 1:100 | 1:50 |
| **3.** | Rabbit anti human calpastatin polyclonal antibodies (Thermo Sci.) | 1:100 | 1:50 |
| **4.** | Mouse anti mouse calpastatin monoclonal antibodies (Thermo Sci.) | 1:1000 | 1:250 |
| **5.** | Rabbit anti bovine HMWCaMBP polyclonal antibodies (in house - crude) | 1:25 | 1:12.5 |
| **Secondary antibodies** | | | |
| **1.** | Goat anti mouse IgG/M polyclonal antibodies – FITC conjugated (Jackson ImmunoResearch) | 1: 400 |  |
| **2.** | Goat anti mouse IgG/M/A polyclonal antibodies – Alexa Fluor 488 conjugated (Life Technologies) |  | 1:1000 |
| **3.** | Goat anti mouse IgG polyclonal antibodies – PE conjugated (Thermo Sci.) |  | 1:400 |
| **4.** | Goat anti rabbit IgG polyclonal antibodies – Alexa Fluor 488 conjugated (Life Technologies) |  | 1:500 |
| **5.** | Goat anti rabbit IgG polyclonal antibodies – PE conjugated (Thermo Sci.) | 1: 300 | 1:750 |
