# Supplementary material for: Ischemia and Reperfusion Induce Differential Expression of Calpastatin and Its Homologue High Molecular Weight Calmodulin-Binding Protein in Murine Cardiomyocytes
Source: PLoS One. 2014 Dec 8;9(12):e114653. doi: 10.1371/journal.pone.0114653 (PMC4259361; doi:10.1371/journal.pone.0114653)
Supplement: S1 Methods — Document describing methodology not provided in the manuscript. (DOC) [file pone.0114653.s008.doc]

**Supplementary Methods**

**Induction of ischemia and reperfusion (I/R)**

The media in NMCC cultures (~80% confluent) was replenished 24 hrs before the induction. Ischemia was induced by replacing the media with a nutrient deficient buffer (NDB) for 2 hrs. NDB was added to the cells for induction and incubated at various time periods for the initial optimization (1, 2 and 4 hrs) [1]. NDB contains 136 mM NaCl, 5 mM KCl, 1 mM CaCl_2_, 0.5 mM MgCl_2_.7H_2_O and 5.5 mM HEPES (pH-6.8) [2,3]. Prior to inducing ischemia in NMCC, glucose and FCS were added to NDB to obtain a final concentration of 5 mM and 2%, respectively [4]. Following induction, NDB was removed and the cells were immediately reperfused or were used for analysis. Reperfusion was carried out by replacing NDB with a standard growth media. In addition, to emulate the oxidative stress in cardiomyocytes observed *in vivo* during reperfusion, hydrogen peroxide (H_2_O_2_) was added to the standard growth media (1 mM final concentration) [1,2,4]. The cells were incubated with this media for 2 hrs prior to analysis. Control cultures (untreated) were maintained in standard cardiomyocyte maintenance media used for NMCC.

**Assessment of protein expression and viability**

Simultaneous assessment of protein expression in control, ischemic and reperfused cardiomyocytes along with viability was performed by FACS. The methodology was performed as per a previously published protocol [1]. The antibodies used have been tabulated along with dilutions used in Supplementary Table S1.

In brief, the medium was removed from the control, ischemic and reperfused cells, which were then washed with Dulbecco's Phosphate-Buffered Saline (DPBS). As suggested by the manufacturer, 7-AAD staining solution in DPBS (~0.25 μg/10^6^ cells) was incubated with control, ischemia and reperfusion induced cells for 10 mins at room temperature in the dark. The cells were washed twice with DPBS and dislodging for FACS. The cells were trypsinized with 0.25% trypsin for 2-3 mins since; the proteins to be studied are intracellular in nature. Dislodged cells were neutralized with media containing 20% FCS, pelleted at 300xg for 5 mins and then washed with DPBS twice. The cell count was performed with a Neubauer counting chamber and ~1 x 10^6^ cells were aliquoted into FACS tubes before fixation in 4% paraformaldehyde in DPBS for 20 mins at room temperature. Cells were rinsed with DPBS twice and incubated in 0.25% Triton-X 100 in DPBS for 10 mins on ice. The cells were pelleted and washed with DPBS twice and then incubated in a blocking solution containing 5% goat serum in DPBS for 1 hr on ice. Cells were pelleted at 600xg for 5 mins and washed with DPBS. The cells were then incubated with primary antibodies against cardiac proteins diluted in DPBS with 0.25% goat serum at varying amounts (see Supplementary Table 1) for 1 hr on ice with intermittent shaking. After incubation, cells were washed with DPBS twice and further incubated with the secondary antibodies diluted in DPBS with 0.25% goat serum for 1 hr on ice in dark with intermittent shaking. The stained cells were washed three times with DPBS and stored at 4°C in the dark in DPBS with 0.2% goat serum. Prior to analysis, the cells were pelleted and resuspended in DPBS. Cell suspension was filtered using a 40 μm cell strainer into labeled FACS tubes. FACS analysis was performed on a flow cytometer (Beckman Coulter) using 488nm (blue) excitation laser and 20,000 events recorded for each sample. The FL-1 channel detected FITC (520 nm), FL-2 detected PE (575nm) and FL-3 was used to detect 7-AAD stain (675nm). The data was then analyzed offline with Flowjo™ software.

**Microscopy**

Fluorescent and subsequently confocal microscopy was performed to demonstrate the protein expression in control (untreated), ischemic and reperfused cardiomyocytes. The methodology was initially optimized by staining cells with primary antibodies and subsequently with the appropriate fluorophore conjugated secondary antibodies and was observed under a fluorescent microscope. The antibodies used for the study have been tabulated along with the dilutions used in Supplementary Table S1.

In brief, the cardiomyocytes were grown on 24 well plates (precoated with 0.2% gelatin) for optimization with a normal fluorescent microscope. Confocal Microscopy was performed on cardiomyocytes grown in 0.2% gelatin precoated 8 well staining slides (Ibidi). The slides and plates were appropriately labeled and treatments (ischemia and reperfusion induction) were performed as described in the Materials and methods section. Following treatment the wells were carefully washed with DPBS twice. The cells were fixed and permeabilized for 20 min. at 4°C using BD cytofix/cytoperm fixation/permeabilization kit as per the manufacturer’s protocol (BD Biosciences) [5]. Fixed/permeabilized cells were then stained with primary antibodies diluted in BD Perm/Wash buffer along with appropriate negative control and incubated at 4°C for 30 min in the dark. 1% Goat serum (Life Technologies) was added to prevent non-specific staining. Following a double wash with BD Perm/Wash buffer, the cells were again stained with antibodies diluted in BD Perm/Wash buffer along with appropriate negative control and incubate at 4°C for 30 min in the dark. The wells were again washed with BD Perm/Wash buffer and then washed once with PBS. Excess liquid from the well was removed by tapping the side of the slide or plate on to a clean laboratory wipe. 1 drop of SlowFade® Gold antifade reagent with DAPI (Life Technologies) was added on the specimen and the samples were cured as suggested by the manufacturer. Following 24 hrs of curing time at room temperature in dark, the samples were stored at 4°C before microscopy. Stacks of images were acquired by optical sectioning cells every 0.75μm with a confocal microscope (Carl Zeiss) using a 20X objective. Saturation was avoided by adjusting photomultiplier sensitivity.

**References**

1. Parameswaran S, Sharma RK (2014) Altered expression of calcineurin, calpain, calpastatin and HMWCaMBP in cardiac cells following ischemia and reperfusion. Biochem Biophys Res Commun 443: 604-609.
2. Haas S, Jahnke H-G, Moerbt N, von Bergen M, Aharinejad S, et al. (2012) DIGE Proteome analysis reveals suitability of Ischemic Cardiac *In Vitro* Model for studying cellular response to Acute Ischemia and Regeneration. PLoS ONE 7: e31669.
3. Li HZ, Guo J, Gao J, Han LP, Jiang CM, et al. (2011) Role of dopamine D2 receptors in ischemia/reperfusion induced apoptosis of cultured neonatal rat cardiomyocytes. J Biomed Sci 18: 18.
4. Pikkarainen S, Kennedy RA, Marshall AK, Tham EL, Lay K, et al. (2009) Regulation of Expression of the Rat Orthologue of Mouse Double Minute 2 (MDM2) by H2O2-induced Oxidative Stress in Neonatal Rat Cardiac Myocytes. J Biol Chem 284: 27195-27210.
5. Sawant A, Schafer CC, Jin TH, Zmijewski J, Tse HM et al. Enhancement of antitumor immunity in lung cancer by targeting myeloid-derived suppressor cell pathways. Cancer Res 2013;73:6609-20.
